# Supplementary material for: The Frequency of Primary Healthcare Contacts Preceding the Diagnosis of Lower-Extremity Arterial Disease: Do Women Consult General Practice Differently?
Source: J Clin Med. 2022 Jun 24;11(13):3666. doi: 10.3390/jcm11133666 (PMC9267865; doi:10.3390/jcm11133666)
Supplement: Supplementary file 1 [file jcm-11-03666-s001.zip › jcm-1732155-supplementary.pdf]

Supplementary material

Table S1: number of GP contacts six months prior to the index date

Number of health care contacts	PAD cohort		Reference cohort		Entire cohort	
	Women (%)	Men (%)	Women (%)	Men (%)	Women (%)	Men (%)
0	545 (31.0)	687 (30.1)	2646 (54.5)	3367 (60.0)	3191 (48.3)	4054 (51.2)
1	195 (11.1)	255 (11.2)	499 (10.3)	544 (9.6)	694 (10.5)	799 (10.1)
2	196 (11.1)	232 (10.2)	427 (8.8)	434 (7.7)	623 (9.4)	666 (8.4)
3	156 (8.8)	194 (8.5)	333 (6.9)	334 (5.9)	489 (7.4)	528 (6.7)
4	107 (6.1)	183 (8.0)	227 (4.8)	254 (4.5)	334 (5.1)	437 (5.1)
5	83 (4.7)	145 (6.3)	157 (3.2)	175 (3.1)	240 (3.6)	320 (4.0)
6	80 (4.5)	99 (4.3)	134 (2.8)	125 (2.2)	214 (3.2)	224 (2.8)
7	65 (3.7)	106 (4.6)	95 (1.9)	95 (1.7)	160 (2.4)	201 (2.5)
8	49 (2.8)	63 (2.7)	69 (1.4)	69 (1.2)	118 (1.8)	132 (1.7)
9	45 (2.5)	56 (2.4)	59 (1.2)	48 (0.9)	104 (1.6)	104 (1.3)
10	50 (2.8)	54 (2.4)	48 (1.0)	40 (0.7)	98 (1.5)	94 (1.2)
>10	190 (10.8)	209 (9.1)	157 (3.2)	150 (2.7)	347 (5.2)	359 (4.5)
**Total**	**1761**	**2283**	**4851**	**5635**	**6612**	**7918**
Minimum	0	0	0	0	0	0
Maximum	79	57	35	66	79	66
Mean	4.06	3.97	1.94	1.67	2.50	2.33
SD	5.44	5.17	3.39	3.32	4.15	4.08
Median	2	2	0	0	1	0
IQR	6	6	3	2	3	3
Variance	29.59	26.72	11.49	11.02	17.22	16.64
Skewness	3.29	2.74	2.95	4.58	3.49	3.71

LEAD: lower extremity arterial disease
SD: standard deviation
IQR: interquartile range

Table S2: Interaction regression model in the PAD and reference cohort

	LEAD cohort				Reference cohort			
	**Negative binomial model[1] (Count model)**		**Zero-inflated model[2] (Logit model)**		**Negative binomial model[1] (Count model)**		**Zero-inflated model[2] (Logit model)**	
Predictors	*Exp (β)**	*CI*	*Exp (β)***	*CI*	*Exp (β)**	*CI*	*Exp (β)***	*CI*
**Intercept[+]**	**2.77**	**2.36 – 3.25**	**2.45**	**1.68 – 3.56**	**1.53**	**1.36 – 1.72**	**6.86**	**5.45 – 8.62**
Sex (men)	0.91	0.75 – 1.12	1.20	0.72 – 2.01	1.21	1.04 – 1.41	1.18	0.87 – 1.60
Diabetes mellitus	1.93	1.72 – 2.16	0.08	0.02 – 0.23	2.02	1.84 – 2.21	0.01	0.00 – 0.14
Hypertension	1.20	1.05 – 1.38	0.14	0.09 – 0.24	1.48	1.34 – 1.65	0.06	0.04 – 0.09
Hyperlipidemia	1.08	0.96 – 1.21	0.43	0.22 – 0.84	1.11	1.01 – 1.23	0.14	0.08 – 0.25
Musculoskeletal	1.04	0.92 – 1.17	0.36	0.24 – 0.56	1.12	1.03 – 1.23	0.34	0.26 – 0.45
Rheumatic disease	1.12	0.90 – 1.38	0.55	0.20 – 1.49	1.33	1.12 – 1.58	0.16	0.07 – 0.38
Vascular disease[3]	1.19	1.03 – 1.39	0.38	0.12 – 1.19	1.15	1.01 – 1.31	0.10	0.03 – 0.39
MI[4]	1.19	1.01 – 1.39	0.17	0.03 – 0.96	1.41	1.21 – 1.64	0.05	0.01 – 0.31
Tobacco abuse[5]	1.13	1.00 – 1.28	0.68	0.43 – 1.09	1.15	1.00 – 1.31	0.38	0.24 – 0.61
Age[6]	1.01	1.00 – 1.01	0.98	0.97 – 0.99	1.00	1.00 – 1.01	0.96	0.95 – 0.97
Sex * Diabetes mellitus	0.86	0.75 – 1.00	0.31	0.04 – 2.45	0.98	0.86 – 1.11	1.47	0.04 – 48.97
Sex * Hypertension	0.99	0.83 – 1.17	0.58	0.26 – 1.30	0.80	0.69 – 0.91	0.97	0.59 – 1.59
Sex * Hyperlipidemia	0.99	0.84 – 1.15	0.63	0.24 – 1.63	0.95	0.83 – 1.09	2.45	1.23 – 4.88
Sex * Musculoskeletal	1.07	0.92 – 1.25	1.04	0.57 – 1.88	0.92	0.81 – 1.04	1.02	0.70 – 1.49
Sex * Rheumatic disease	0.92	0.65 – 1.31	1.22	0.14 – 10.84	0.86	0.66 – 1.12	1.17	0.31 – 4.49
Sex * Vascular disease[3]	0.99	0.81 – 1.20	0.21	0.03 – 1.61	1.08	0.90 – 1.30	2.28	0.52 – 9.92
Sex * MI[4]	1.04	0.86 – 1.27	0.50	0.06 – 3.98	0.80	0.66 – 0.97	0.82	0.10 – 6.71
Sex * Tobacco abuse[5]	1.14	0.97 – 1.33	0.85	0.43 – 1.66	1.07	0.89 – 1.28	0.91	0.48 – 1.71
Sex * Age[6]	1.00	0.99 – 1.01	1.00	0.98 – 1.02	1.00	0.99 – 1.00	1.00	0.99 – 1.02

CI: 95% confidence interval

+ The intercept refers to a woman with mean age, the other exponent betas for the different predictor and interactions should be interpreted as factors.

* Exponent beta in the negative binomial part of the model is interpreted as a count

** Exponent beta in the zero inflated (logit model) is interpreted as an odd ratio

[1] Coefficients for the count part of the model are interpreted as predicted number of health care contact

[2] The logistic part of the model predicts non-ocurrence of health care contact

[3]Vascular disease is defined as ICPC codes K89 (Transient ischemic attack) and K90 (Stroke)

[4] History of miocardial infarction

[5] History of tobacco abuse was defined as patients with ICPC code P17 at baseline

[6] Age was mean centred for all analyses.
